# Optimization of the left ventricle ejection fraction estimate obtained during cardiac adenosine stress ^82^Rubidium-PET scanning: impact of different reconstruction protocols

**DOI:** 10.1007/s12350-022-02946-1

**Published:** 2022-04-12

**Authors:** Martin Lyngby Lassen, Mads Wissenberg, Christina Byrne, Andreas Kjaer, Philip Hasbak

**Affiliations:** 1 Department of Clinical Physiology, Nuclear Medicine and PET and Cluster for Molecular Imaging, Copenhagen University Hospital - Rigshospitalet and Department of Biomedical Sciences, University of Copenhagen, Section 4011, Blegdamsvej 9, 2100 Copenhagen, Denmark; 2 Department of Cardiology, Copenhagen University Hospital - Gentofte, Hellerup, Denmark

**Keywords:** ejection fraction, adenosine, PET, cardiac imaging

## Abstract

**Background:**

Left ventricular ejection fraction (LVEF) estimation using adenosine stress myocardial perfusion imaging (MPI) can be challenging. The short half-life of adenosine and the guideline-recommended adenosine infusion stop during Rubidium-82 acquisition protocol may affect the accuracy and repeatability of the LVEF measures.

**Methods:**

This study comprised 25 healthy volunteers (median age 23 years) who underwent repeat myocardial perfusion imaging (MPI) sessions employing Rubidium-82 PET/CT. A guideline-recommended reconstruction protocol was used for both rest and adenosine stress MPI (150-360 s post-radiotracer injection, standard_recon_). For the stress MPI protocol, two additional reconstruction protocols were considered; one was employing 60 seconds data (150-210 seconds, short_fixed_) and the other a dynamic frame window based on the bolus arrival of Rubidium-82 in the heart until 210 seconds (*x*-210 seconds, short_individual_). We report rest and stress LVEF, the LVEF reserve, and the LVEF reserve repeatability.

**Results:**

Differences in the LVEF assessments were observed between the guideline recommended and alternative reconstruction protocol (LVEF stress MPI: standard_recon_ = 68 ± 7%, short_fixed_ = 71 ± 7% (*P* = .08), short_individual_ = 72 ± 7% (*P* = .04)), and the LVEF reserve was reduced for the guideline-recommended protocol (standard_recon_ = 7.8 ± 3.5, short_fixed_ = 10.1 ± 3.7, short_individual_ = 10.5 ± 3.6, all *P *< .001). The best repeatability measures were obtained for the short_individual_ protocol (repeatability: standard_recon_ = 45.3%, short_fixed_ = 41.2%, short_individual_ = 31.7%).

**Conclusion:**

We recommend using the short_individual_ reconstruction protocol for improved LVEF repeatability and reserve assessment. Alternatively, in centers with limited technical support we recommend the use of the short_fixed_ protocol.

**Supplementary Information:**

The online version contains supplementary material available at 10.1007/s12350-022-02946-1.

## Introduction

Rubidium-82 (^82^Rb) myocardial perfusion imaging (MPI) has become a central element in the clinical assessment of myocardial perfusion and function. Myocardial assessment employing ^82^Rb positron emission tomography/computed tomography (PET/CT) offers a versatile assessment of both myocardial perfusion and the left ventricular ejection fraction (LVEF), with the latter providing valuable information on myocardial function.^1–5^ It has been established that increases in the LVEF > 5% from rest to stress MPI (LVEF reserve) have a strong negative predictive value of coronary artery disease.^5,6^ In this context, several pharmacological stressors (adenosine, dipyridamole, and regadenoson) are currently employed in the stress MPI assessments in PET.^3^ Each of these stressing agents prescribes different guideline recommendations for injection and injection-to-scan delays to obtain maximum hyperemic response during the stress MPI sessions.^7,8^ Current guideline recommendations permit full hyperemic response during the MPI when dipyridamole and regadenoson are employed; thus, the LVEF increase reflects the maximum hyperemic response.^7,8^ Common for all the adenosine protocols is the termination of the infusion midway into the PET image acquisition to ensure maximum hyperemic response during the perfusion assessment. However, the termination of adenosine midway into the PET acquisition poses the risks of reducing the stress LVEF estimates owing to the short biological half-life of adenosine (~ 1-10 seconds^9^).

As the LVEF from PET has become more widely used in a clinical setting, focusing on its accuracy also becomes increasingly important. With this study, we aimed to evaluate how different reconstruction windows affect the LVEF assessments for stress MPI. Six stress MPI reconstruction protocols with different reconstruction windows were used, either based on fixed time windows (three reconstruction protocols) or suited to the individual MPI sessions (three reconstruction protocols).

## Materials and methods

### Study population

This study comprised 25 young, healthy volunteers (11 females) (median age = 23 years (interquartile range (IQR) = [22; 25])) recruited for rest/adenosine stress myocardial perfusion ^82^Rb-PET/CT. Median volunteer weight was 70.0 kg [IQR = 62; 79.5 kg], with corresponding median BMI on 22 [IQR = 20.5; 23.8]. The volunteers underwent repeat PET/CT imaging sessions within 27 days [IQR = 17; 36]. Inclusion criteria were age >18 years, no regular consumption of medicine, no known medical conditions, and no use of tobacco and euphoric substances (except alcohol) within three months prior to study participation. Exclusion criteria were pregnancy, allergy or intolerance to theophylline or adenosine, any prior medical history of asthma, or inability to adhere to the study protocol. The Scientific Ethics Committee of the Capital Region of Denmark [protocol number H-15009293] and the Danish Data Protection Agency approved this study.

### Imaging protocol

#### PET acquisition

**Figure 1:**
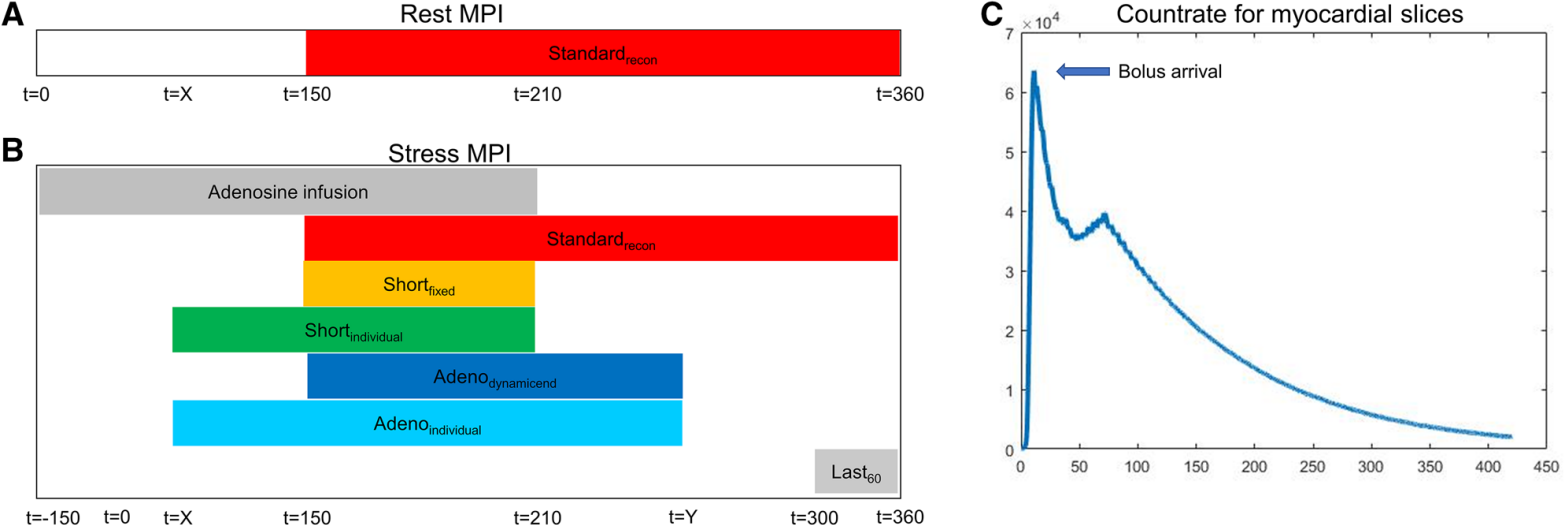
Reconstruction protocol. Reconstruction protocol for the rest (A) and stress (B) MPI. Standard_recon_ protocols contain data from 210 seconds, starting at 150 seconds post-^82^Rb injection, and short_fixed_ contains 60 seconds data ranging from 150 to 210 seconds (end of adenosine infusion). Short_individual_ has a bolus-dependent reconstruction time (denoted as *x*) and a data collection end time point of 210 seconds, with the bolus arrival as determined by the count rate peak for the myocardial slices + 90 seconds (C). Specific for the stress MPI sessions, the “Adeno” reconstructions both have end of data collection for the ECG-gated reconstructions based upon the end of the hemodynamic response (denoted as *y*), where Adeno_dynamicend_ has a fixed start (150 seconds) and the Adeno_individual_ has a bolus-dependent start of data collection. *MPI*, myocardial perfusion imaging

The 25 healthy volunteers underwent repeated ^82^Rb-PET/CT MPI sessions. All MPI sessions consisted of an ^82^Rb rest/stress protocol targeting injections of 1100MBq (30mCi) obtained on a 128 slice Siemens Biograph mCT PET/CT system (*5*). Pharmacological stressing was obtained using adenosine infused at 140mg/kg/min for 6 minutes with PET emission acquisition starting 2.5 min into the infusion (Figure 1). Each imaging session started with a low-dose CT for attenuation correction purposes acquired using a free-breathing protocol,^10^ followed by the PET emission scans. The volunteers were instructed to abstain from caffeine at least 24 hours before each of the imaging sessions.

#### PET reconstruction protocol

The PET acquisitions were reconstructed into eight cardiac phases (8-ECG gates) using a single reconstruction protocol for rest MPI and six different reconstruction protocols for stress MPI. All reconstruction protocols employed two iterations and 21 subsets (Ordered subset expectation maximization) with corrections for point spread function and time of flight, followed by a 5 mm Gaussian filtering.^11^ For both rest and stress MPI, a guideline-recommended reconstruction protocol (eight phases, using data obtained between 150 and 360 seconds into the PET acquisition) was considered (Figure 1). For the stress MPI, the additional five reconstruction protocols aimed at optimizing the reconstruction protocol to compensate for the rapid metabolism of adenosine. The first two data series focused on ending the reconstruction window at the end of adenosine infusion (210 seconds), using either a fixed starting point (150 seconds) based on the standard reconstruction windows (150-210 seconds, short_fixed_) or an individual start of the reconstruction window as determined by the ^82^Rb bolus arrival to the myocardium (*x*-210 seconds, short_individual_) (^*82*^*Rb bolus detection).* The next two reconstruction protocols had a reconstruction window end determined by the end of the hyperemic response, which was defined by the changes in the heart rate (*Stress MPI: End of hyperemic response*). The first focused on a fixed beginning of the reconstruction window (150 seconds) as determined by the standard reconstruction protocol (150-*x* seconds) (Adeno_dynamicend_), while the second employed an individual reconstruction window start and end time (*x*-*x*s) (Adeno_individual_). Finally, a reconstruction employing data from 300 to 360 seconds post-radiotracer injection was evaluated to test the effect of adenosine metabolism (Figure 1).

#### ^82^Rb bolus detection

The ^82^Rb bolus arrival was determined using the PET raw data (listmode data) from which sinograms were generated for every 200 ms.^12,13^ Utilizing a myocardial segmentation from the CT attenuation correction maps, the bolus arrival to the heart was estimated from count rate assessments (Figure 1C). A delay of 90 seconds from detecting the bolus in the myocardial plane was introduced to allow for sufficient blood-pool clearance^14^ (Figure 1).

#### Stress MPI: End of hyperemic response

The end of the hyperemic response was estimated from analyses of the heart rate as obtained from the ECG-trigger events (3-lead ECG triggering). The average heart rate obtained during the rest MPI was used to estimate the approximate baseline heart rate. The hyperemic response (stress MPI) was assumed to have ended when the heart rate during the stress MPI returned to the baseline heart rate ± .3 standard deviations following the end of the adenosine infusion (210 seconds into the stress MPI) (Figure 1).

### Data analysis

Assessment of bolus arrival and the end of the hemodynamic response was calculated in custom-made software (MatLab, Mathworks). We report rest and stress stroke volume (SV), the end-diastolic volumes (EDV), LVEF, as well as the LVEF reserve (stress LVEF-rest LVEF) for all participants. All EF measurements were calculated using Cedars QPS (Cedars-Sinai). Further, we report the %-wise count rates and random events employed for the proposed reconstructions, normalized to the stress standard_recon_ protocol.

### Statistical analysis

For descriptive analyses of continuous values, we used mean ± standard deviation, range or median, and interquartile range. Changes in the volumetric analyses were tested for differences using one-way analysis of variance (one-way ANOVA) using Tukey-Kramer test for differences results obtained from the respective reconstruction protocols. All one-way ANOVA’s were considering the repeated measurements and performed in R (the GNU project). Two-tailed *P *values less than .05 were considered statistically significant. All data were checked for normality using Shapiro-Wilk test. Test-retest repeatability is calculated using the RMS method^15^:

1 $$RMS\left(\%\right)=100\times \sqrt{\frac{\sum {\left(\frac{d}{m}\right)}^{2}}{2n}}$$

In the equation, *m* represents the mean of the paired measurements, *d* is the difference between the two paired measurements, and *n* is the sample size.

## Results

### Heart rate assessment, reconstruction duration, and count rates

**Figure 2:**
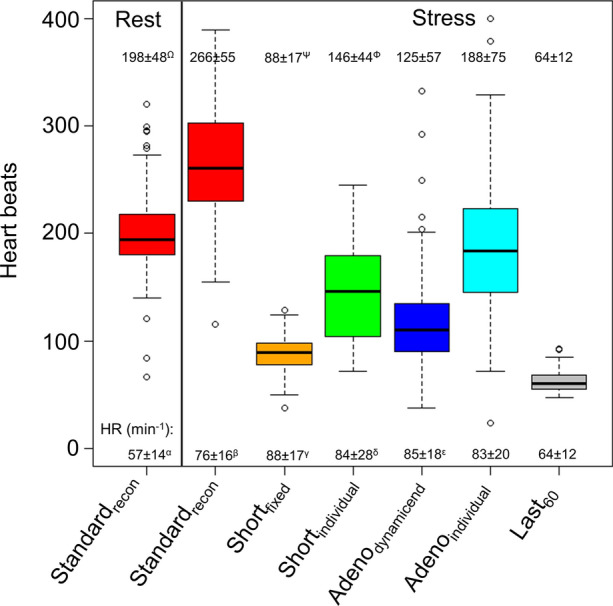
Number of heartbeats obtained for the reconstructions. A minimum of 38 beats was included for the reconstructions (short_fixed_, rest study), while a maximum of 400 beats was included (Adeno_individual_, Stress MPI) and, thus, sufficient count rates were ensured for the reconstructions for all reconstructions. Of note, the heart rate (HR) is shown at the bottom for the respective reconstruction protocols. Significant differences were observed between all the reconstruction protocols, with markings indicating non-significant differences between a subset of the reconstructions. *MPI*, myocardial perfusion imaging. Non-significant differences were observed between the following number of heart beats: ^Ω^rest Standard_recon_ and Adeno_individual_, ^Ψ^short_fixed_ and Last_60_, and ^Φ^short_individual_ and adeno_dynamicend._ Non-significant differences were observed between the following number of heart rates: ^α^rest Standard_recon_ and Last_60_, ^β^stress standard_recon_ and short_individual_, adeno_dynamicend,_ and adeno_individual_, ^γ^short_fixed_ and short_individual_, adeno_dynamicend,_ and adeno_individual_, ^δ^short_individual_ and adeno_dynamicend_ and adeno_individual_, and ^ε^adeno_dynamicend_ and adeno_individual_

All six (stress MPI) reconstruction protocols had significant differences in the duration of the reconstruction frames obtained, with average reconstruction durations ranging from 57 to 210 seconds (all *P *< .001). Despite significant variations in the duration of reconstruction windows, sufficient data were collected for all reconstructions with a minimum number of heartbeats employed of 38 (Figure 2). Of note, significant changes in the heart rate were observed during the stress MPI sessions, ranging from an average heart rate of 64 to 88 (stress Standard_recon_ had significantly reduced heart rate compared to all proposed stress reconstruction protocols except Last_60_, all *P *< .001). In this context, the average heart rate for the rest scans was 57 (Figure 2). Assessment of count rates revealed significantly reduced prompts and random events for the proposed reconstructions with fixed reconstruction window starting times (short_fixed_, adeno_dynamic_, Last_60_) compared to the standard_recon_ (Supplementary Figure 1). In comparison, significantly increased prompts and random events are reported for the datasets with dynamic reconstruction window starting points (short_individual_, adeno_individual_) (Table 1)

### Quantitative measures

**Figure 3:**
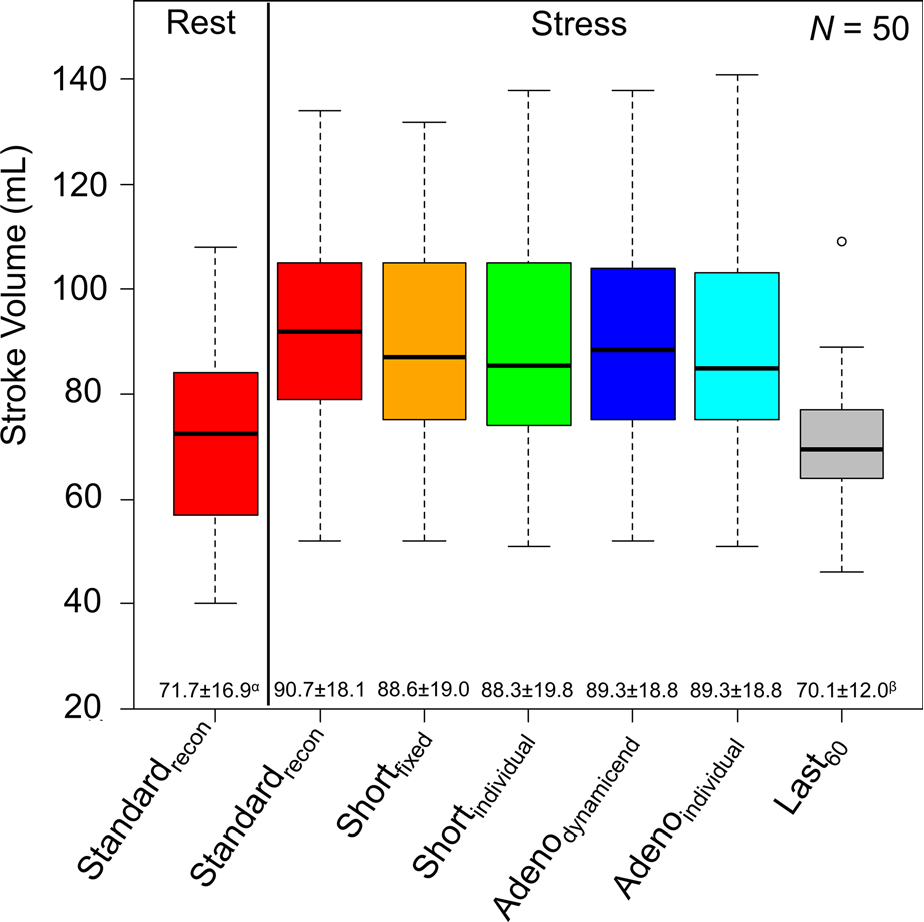
Stroke volume obtained for the six reconstruction protocols. Mean and standard deviations obtained for the respective protocols are shown at the bottom row. No differences were observed between the reconstruction protocols, except for the reconstructions protocols with markings where differences to one or more protocols are reported. Significant differences were observed between the following SV: ^α^rest Standard_recon_ and all stress reconstruction protocols except Last_60_, ^β^Last_60,_ and all other stress reconstruction protocols

Assessments of the SV revealed comparable measures for all stress MPI sessions except for the Last_60_ reconstructions (all *P* ≥ .99) (Figure 3). All stress reconstructions, except the Last_60_ reconstruction, also had significantly increased SV when compared to the SV obtained from the rest acquisition (all *P *< .001) (Figure 3).

**Figure 4:**
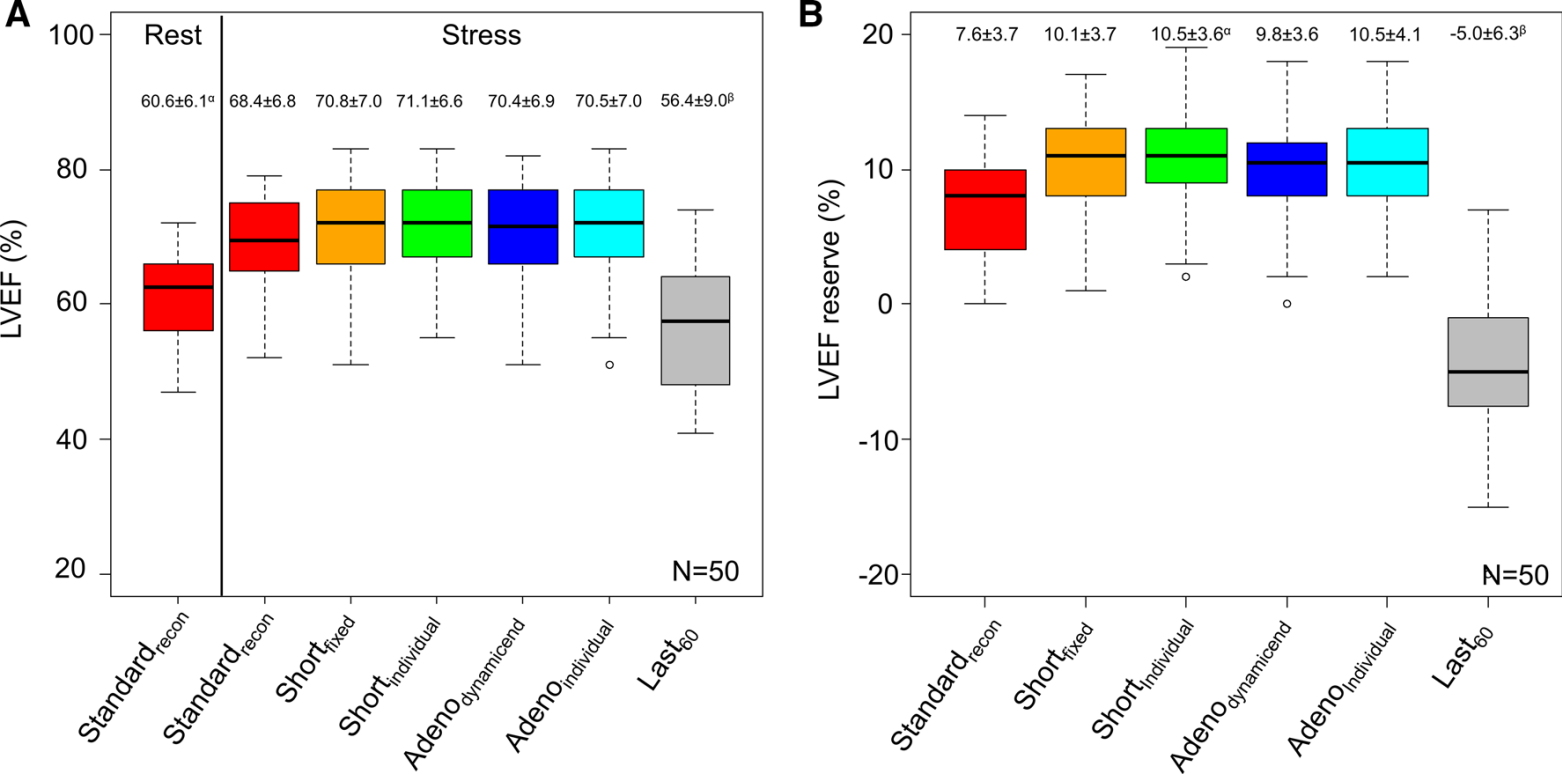
LVEF and LVEF reserve observed for the volunteers reported as the empirical measurements and the percentwise increase. No differences were observed between the reconstruction protocols, except for the reconstructions protocols with markings where differences to one or more protocols are reported. Significant differences were observed between the following LVEF (**A**): ^α^rest Standard_recon_ and all stress reconstruction protocols except Last_60_, ^β^Last_60,_ and all other stress reconstruction protocols. Significant differences were observed between the following LVEF reserves (**B**): ^α^short_individual_ and stress standard_recon_, ^β^Last_60,_ and all other stress reconstruction protocols

**Figure 5:**
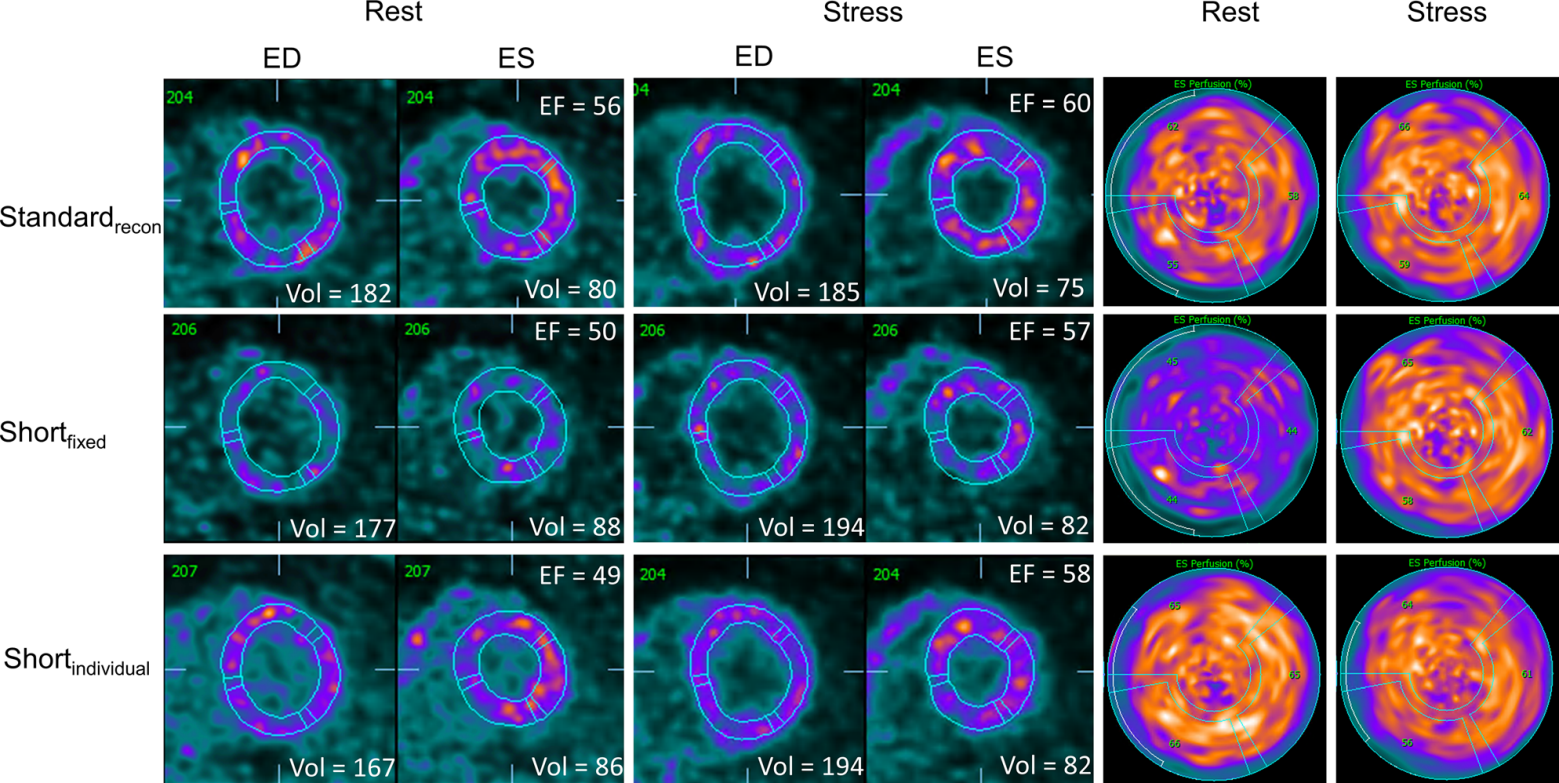
Case example. Test subject with LVEF increase <5% from rest to stress MPI for Standard_recon_, while LVEF increase ≥ 5% for the proposed protocols. Similar uptake patterns are shown for the standard_recon_ and the short_individual_ reconstruction protocols, while reduced contrast is observed for the short_fixed_. *ED*, end diastolic; *ES*, end systolic; *EF* ejection fraction; *Vol*, left ventricular wall volume

**Figure 6:**
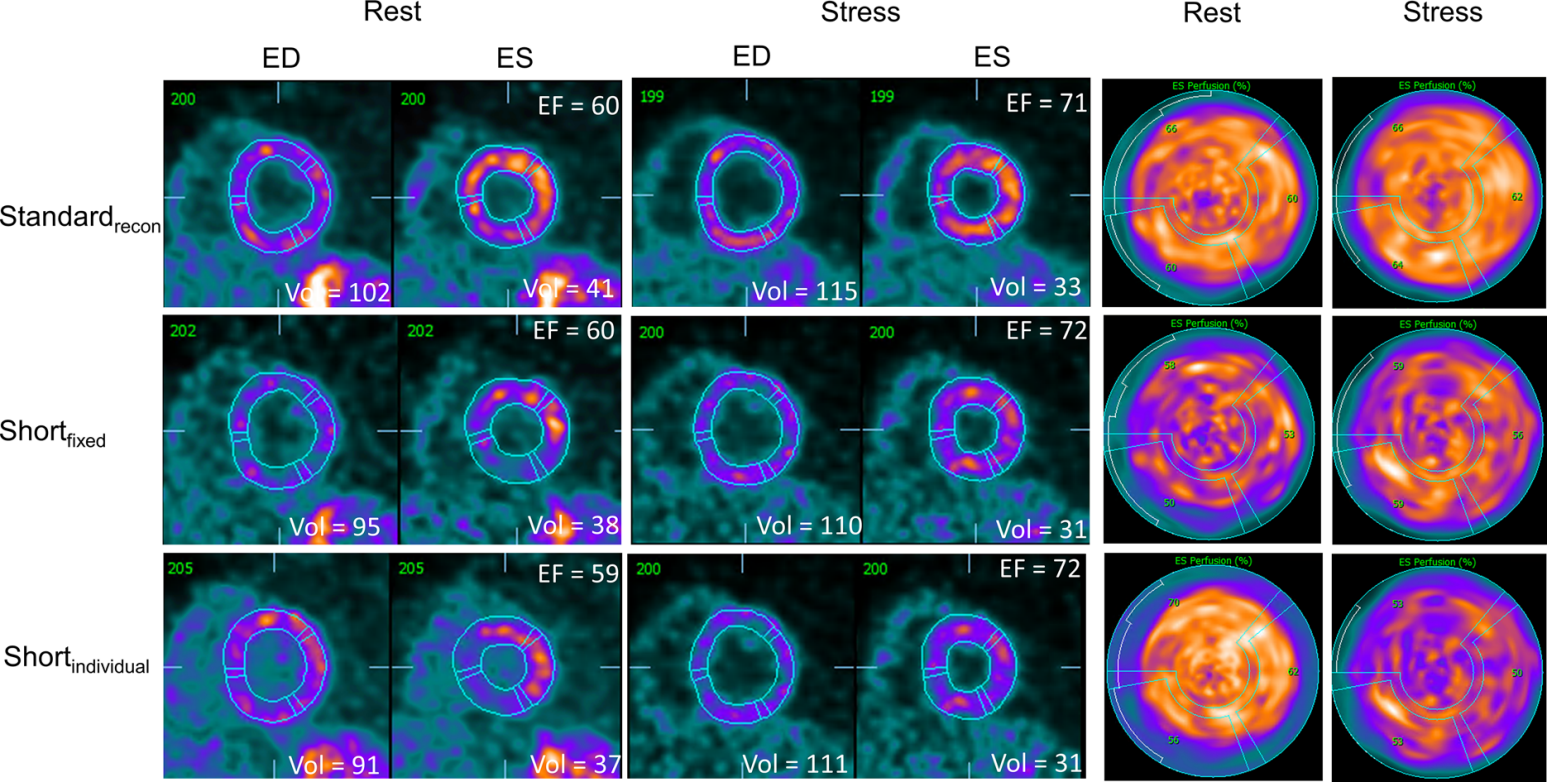
Case example. Homogeneous volumetric measurements were obtained for all three reconstruction protocols. *ED*, end diastolic; *ES*, end systolic; *EF*, ejection fraction; *Vol*, left ventricular wall volume

Assessments of the LVEF revealed similar trends for the proposed reconstruction protocols as observed for the SV assessments (rest LVEF vs all stress protocols, except Last_60_: *P *< .001, Last_60_ vs all other stress reconstruction protocols: *P *< .001, and rest LVEF vs Last_60_: *P* = .053, all stress protocols except Last_60_: *P* ≥ .485) (Figure 4). The highest stress LVEF estimate was reported for the short_individual_ protocol with an average stress LVEF of 71.1%. Similarly, the greatest LVEF reserve was reported for the short_individual_, with an average LVEF reserve of 10.5; in contrast, the Standard_recon_ protocol suggested an average LVEF reserve of 7.6. Of the proposed reconstruction protocols, short_indivdual_ had a significantly improved LVEF reserve when compared to the standard_recon_ (*P* = .023), while short_fixed_ may be considered to have improved LVEF reserve if less stringent criteria for differences are used (*P* = .109). The lowest LVEF reserve was obtained when using the last 60 seconds of data for reconstruction, with an average LVEF reserve of − 5.0. Two case examples are shown in Figures 5 and 6.

### Test-retest repeatability

Significantly improved test-retest repeatability was reported for the short_individual_ reconstruction protocol for the stress LVEF (2.7%) and LVEF reserve (33.9%) when compared to the standard reconstruction protocols (3.5% and 46.7%, respectively) (both *P *< .001) (Table 2).

**Table 1:** Scan duration and counts used for the proposed reconstruction protocols

	Reconstruction duration (s)		Counts employed (normalized to standard, %)
	Rest	Stress	Stress
Standard_recon_	210 ± 0	210 ± 0	100 ± 0
Short_fixed_	NA	60 ± 0*	57 ± 6*
Short_individual_	NA	106 ± 16*	131 ± 47*
Adeno_dynamicend_	NA	90 ± 42*	70 ± 18*
Adeno_individual_	NA	135 ± 43*	152 ± 49*
Last_60_	NA	60 ± 0*	5 ± 1*

Of note, all counts for the proposed reconstruction protocols were normalized to the standard_recon_ stress protocol to account for differences in the injected activities and differences in the photon attenuation in the volunteers

*Denotes significant differences between the respective proposed protocols and the stress standard_recon_ protocols. *P *< .05 were considered statistically significant

**Table 2:** Repeatability measures obtained for SV, LVEF, and percent change in EF between rest and stress MPI

	EDV (mL)	SV (mL)		LVEF (%)		LVEF reserve
	Stress	Rest	Stress	Rest	Stress	Combined
Standard_recon_	3.2	7.8	5.3	4.5	3.5	46.7
Short_fixed_	4.2^α^	NA	7.2^α^	NA	4.6^α^	43.2
Short_individual_	3.6	NA	5.8	NA	2.7	33.9
Adeno_dynamicend_	3.8	NA	7.8^β^	NA	4.7	42.3^α^
Adeno_individual_	3.5^β^	NA	6.6	NA	5.8	53.2
Last_60_	4.6	NA	9.1	NA	7.3	94.2

Test-retest measures were obtained using the RMS method, with the unit being in % repeatability. Statistical differences in the test-retest measures were evaluated using Pitman-Morgan tests, with *P *< .05 indicating statistically significant differences. Significant differences were obtained for all repeatability measures, except the ones indicated with a marker

For EDV, non-significant differences were observed between ^α^short_fixed_ and adeno_dynamicend_ and ^β^adeno_indiviudal_ and standard_recon_ and short_individual_

For SV, non-significant differences were observed between ^α^short_fixed_ and adeno_dynamicend_ and adeno_individual_ and ^β^adeno_dynamicend_ and adeno_individual_

For LVEF, non-significant differences were observed between ^α^short_fixed_ and adeno_dynamicend_

For LVEF, reserve non-significant differences were observed between ^α^adeno_dynamicend_ and stress standardrecon and short_fixed_

## Discussion

This study evaluated the impact of timing and the duration of the reconstruction protocols for ECG-gated studies without and with adenosine stressing in ^82^Rb MPI studies. In this controlled study of healthy young volunteers, we report that the duration of the reconstruction window employed for the rest/stress MPI sessions affects the LVEF reserve assessments and their repeatability measures. The main finding of our study is that the proposed reconstruction protocols, except for Last_60_, unanimously increase the LVEF and LVEF reserve, although only the changes in the LVEF reserve or the short_individual_ were significantly increased. This might suggest that using the guideline-proposed reconstruction protocols may reduce accuracy in the LVEF estimates (reduced test-retest repeatability) and reduce LVEF reserve.

Current guidelines recommend ECG-gated studies to assess LVEF and LVEF reserve as prognostic markers in the detection of CAD.^3^ Studies of LVEF have shown that increments of > 5% in LVEF reserve between rest and stress MPI might suggest left main/3-vessel CAD.^5,16^ However, the LVEF reserve mandates assessment at peak vasodilation, which might be compromised in routine ^82^Rb MPI protocols employing adenosine as a vasodilator. Adenosine has a half-life of .6-10 seconds,^9^ suggesting that current ECG-gated reconstruction protocols might be obtained outside of peak vasodilation. To test this hypothesis, we evaluated six reconstruction protocols for the stress MPI, comparing the guideline-recommended reconstruction protocol to reconstructions obtained in the last minute in addition to four proposed reconstruction protocols. Unanimously, all proposed reconstruction protocols, focusing on employing only data during the maximum hyperemic response, showed elevations in the stress LVEF and LVEF reserve, although only short_individual_ LVEF reserve was significantly increased compared to the standard protocol. Further, short_fixed_ and short_individual_ had significantly improved LVEF reserve test-retest repeatability compared to the standard_recon_ (Table 2). The improved LVEF and LVEF reserve measurements (Figure 4) support the hypothesis that adenosine metabolism affects the quantitative analyses and, thus, the test-retest variation. However, some of the variations in the repeatability measures may be attributed to the variations in the count rates employed for the reconstruction protocols (Supplementary Figure 1). While a trend between the counts employed for the reconstructions and the repeatability measures exists for all reconstruction protocols, an improved repeatability coefficient is reported for the short_individual_ compared to adeno_individual_ despite employing fewer counts. Further supporting the hypothesis that adenosine metabolism affects the quantitative measures is the significant drop in the LVEF and LVEF reserve assessments when data acquired from 360 to 420 seconds are evaluated. Analysis of the heart rate for the guideline-recommended protocols revealed an average increase of 19 beats per minute (*P *< .01) during the stress MPI, whereas increases of up to 31 beats per minute were reported for the proposed reconstruction protocols (proposed stress protocols except Last_60_ vs rest: *P *< .001, short_fixed_ vs stress standard_recon_: *P* = .02). In this context, the average heart rate obtained during the last 60 seconds (360-420) only had an increase of 7 beats per minute and was not different from heart rates obtained during rest (*P* = .56), strongly suggesting that no hyperemic response is present during this phase. Therefore, the findings in this study strongly indicate that the use of the shortened protocols (short_fixed_ and short_individual_) are favorable of the proposed reconstruction protocols and favorable compared to the standard_recon_ protocol. While the number of heart beats employed for the reconstructions are significant reduced when compared to the standard_recon_ protocol (Supplementary Figure 1), the number of counts is sufficient to provide acceptable reconstructions with high repeatability measures (Table 2).

Of note, the ^82^Rb bolus arrival to the heart assessment can be evaluated within 5-15 minutes on a standard PC with parallel computing toolboxes and, thus, does not extend the time spent on the analyses.

In this study, the impact of the reconstruction window was evaluated using 8-ECG gates instead of the usually recommended 16-ECG gates. The decision on using 8-ECG gates was driven by the desire to test the impact of the adenosine metabolism and its potential impact on the LVEF assessments obtained during the 6th minute of the acquisitions as shown in Supplementary Figure 1. Using 16-ECG gates would lead to too few counts (~ 5% of the counts employed for the standard_recon_ protocol, Supplementary Figure 1) being present to obtain reliable segmentation of the left ventricle, which is in concordance with previous suggestions on using 8-ECG-gated reconstructions when employing ^82^Rb.^17^ However, using 8-ECG gates might reduce repeatability measures in patients with low EF, lending that the repeatability measures reported here may be reduced in patients. Despite the potential impact of using too few ECG gates in the assessment of patients with low LVEF, we do not find this a limitation in the current study as only healthy, young volunteers were considered. This limitation, however, should be considered in the analysis of patients with reduced LVEF and other comorbidities.

## Study limitations

This study has several limitations. The LVEF changes observed in this study are probably specific for subjects during adenosine stress. The choice of pharmacologic stress agents is dependent upon patient characteristics and the stress imaging study being performed, as well as institutional and/or provider preference. When using other pharmacological stressors, like dipyridamole, regadenoson, or dobutamine, the hyperemic steady state occurs before the rubidium infusion takes place why the LVEF estimate will probably not be change as much—although not tested in this study. The study only included 25 study subjects, each having repeated MPI sessions. Albeit the low number of subjects included, significant and unanimous results were reported for the proposed reconstruction protocols. Another limitation of the population is that the subjects were healthy volunteers of young age without the comorbidities that would be expected in elderly cohorts. Patients with comorbidities and increased age are expected to have reduced hyperemic response when compared to young volunteers, thus, suggesting that personalized reconstruction protocols might be more advantageous in patients. A follow-up study focusing on patient cohorts is currently being evaluated in our center.

## New knowledge gained

This study evaluated the accuracy and repeatability of LVEF and LVEF reserve when using adenosine as stressing agent. The main finding was a significant increase in LVEF reserve when using shortened reconstruction protocols focusing only on including data during the adenosine infusion, with simultaneous improvements in the test-retest repeatability. LVEF reserve has previously been shown to provide significant independent and incremental value to ^82^Rb MPI for predicting the risk of future adverse cardiac events. Further, we showed a post-stress LVEF reduction to the level of LVEF during rest in the last minute of the ^82^Rb-PET scan (5-6 minutes after ^82^Rb-infusion start) where the pharmacological effect of adenosine had ceased; an observation possible also providing incremental value to the ^82^Rb MPI, which needs further testing. The awareness of potential changes in LVEF and LVEF reserve depending on the choice of reconstruction protocol is of great importance when interpreting clinical data.

## Conclusion

In healthy subjects, we describe physiological LVEF changes during adenosine stress ^82^Rb-PET. Current guideline-recommended reconstruction windows risk underestimating the LVEF and LVEF reserve assessments. The use of short_individual_ provides the most reliable assessments with improved test-retest repeatability of LVEF and LVEF reserve and significant improvements in the LVEF reserve assessments compared to the guideline-recommended protocols. Therefore, we recommend using the short_individual_ reconstruction protocol for LVEF assessments. Alternatively, in centers with limited technical support, we recommend short_fixed_.

## Supplementary Information

Below is the link to the electronic supplementary material.

Supplementary file1 (DOCX 464 kb)

Supplementary file2 (PPTX 293 kb)

Supplementary file3 (TIF 1263 kb)

## Abbreviations

^82^Rb: Rubidium-82
ANOVA: Analysis of variance
EDV: End-diastolic volume
LVEF: Left ventricular ejection fraction
IQR: Interquartile range
MPI: Myocardial perfusion imaging
PET/CT: Positron emission tomography/computed tomography
SV: Stroke volume
