# Supplementary material for: Optimization of the left ventricle ejection fraction estimate obtained during cardiac adenosine stress 82Rubidium-PET scanning: impact of different reconstruction protocols
Source: J Nucl Cardiol. 2022 Apr 12;29(6):3369–78. doi: 10.1007/s12350-022-02946-1 (PMC9834342; doi:10.1007/s12350-022-02946-1)
Supplement: Supplementary file 1 — Supplementary file1 (DOCX 464 kb) [file 12350_2022_2946_MOESM1_ESM.docx]

**Optimization of the left ventricle ejection fraction estimate obtained during cardiac adenosine stress ^82^Rubidium-PET scanning: Impact of different reconstruction protocols**

Martin Lyngby Lassen, PhD^1^, Mads Wissenberg MD, PhD^2^, Christina Byrne, MD, PhD^1^, Andreas Kjaer, MD, PhD, DMSc^1^, Philip Hasbak, MD, DMSc^1^

^1^Department of Clinical Physiology, Nuclear Medicine and PET and Cluster for Molecular Imaging, Copenhagen University Hospital - Rigshospitalet and Department of Biomedical Sciences, University of Copenhagen

^2^Department of Cardiology, Copenhagen University Hospital - Gentofte, Denmark.

^3^Department of Drug Design and Pharmacology, Faculty of Health and Medical Sciences, University of Copenhagen, Copenhagen, Denmark

Corresponding Author:
Martin Lyngby Lassen, PhD, Department of Clinical Physiology, Nuclear Medicine and PET and Cluster for Molecular Imaging, section 4011, Rigshospitalet and University of Copenhagen, Blegdamsvej 9, 2100 Copenhagen, Denmark

Tel: +45 35453520 Fax: +45 35454015

Email: martin.lyngby.lassen@regionh.dk

Short title: Metabolization of adenosine during stress MPI

Word count: 3500

**Supplementary Figure 1:** **Count rates.** Total prompts (A) and total Randoms (B) obtained for the respective gated reconstructions normalized to the standard stress reconstructions. Significant differences were observed between all the reconstruction protocols, with markings indicating non-significant differences between a subset of the reconstructions.


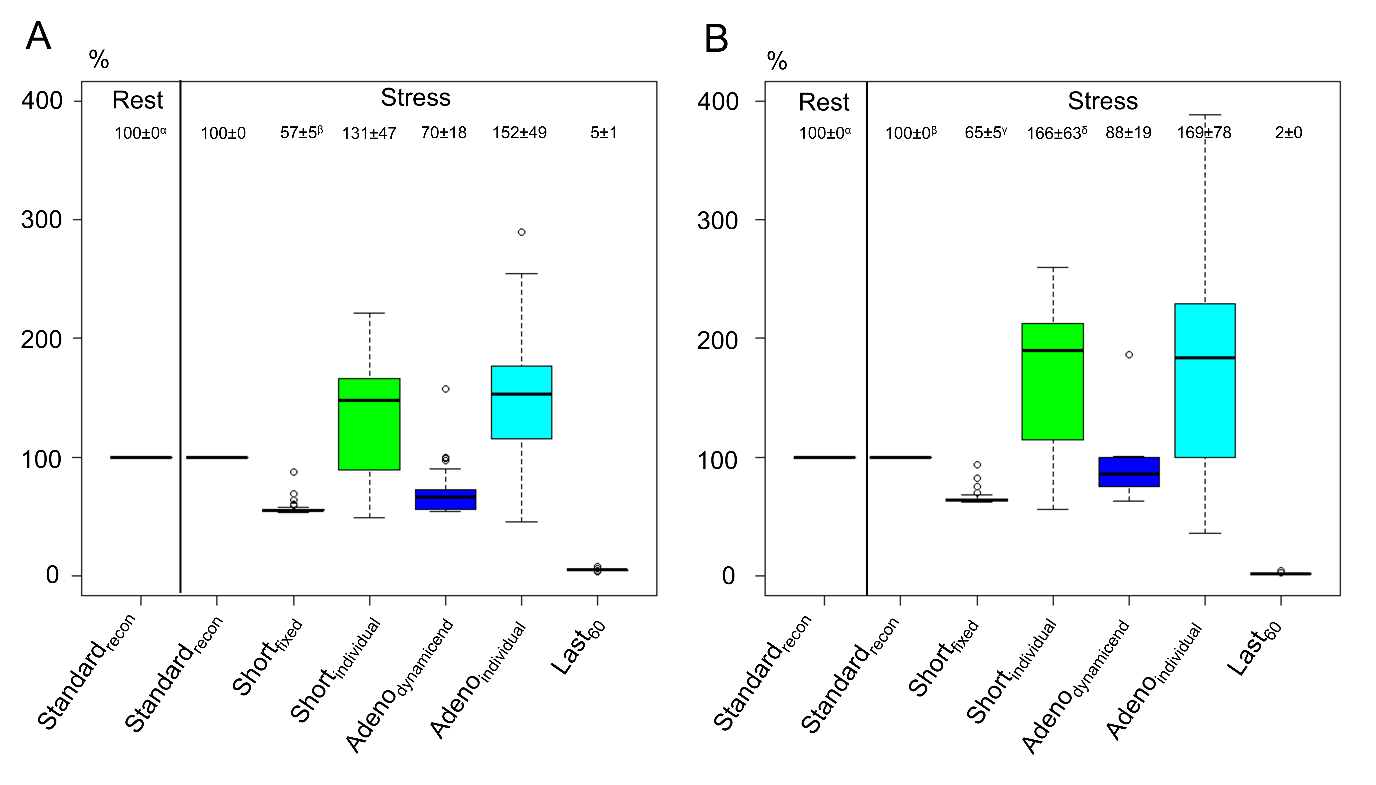


Non-significant differences were observed between the following number of prompts employed for the reconstructions (A): ^α^ = rest Standard_recon_ and stress Standard_recon_, ^β^ = short_fixed_ and adeno_dynamicend._

Non-significant differences were observed between the following number of randoms: ^α^ = rest Standard_recon_ and stress Standard_recon_, and adeno_dynamicend_, ^β^ = stress Standard_recon_, and adeno_dynamicend_, ^γ^ = short_fixed_ and adeno_dynamicend_, ^δ^ = short_individual_ and adeno_individual_

**Supplementary Figure 2:** **EDV measurements.** The EDV were comparable across all reconstructions, with all the stress ECG-gated reconstructions being elevated compared to the datasets obtained in rest. Of note, no differences were reported for any of the reconstruction protocols.

**
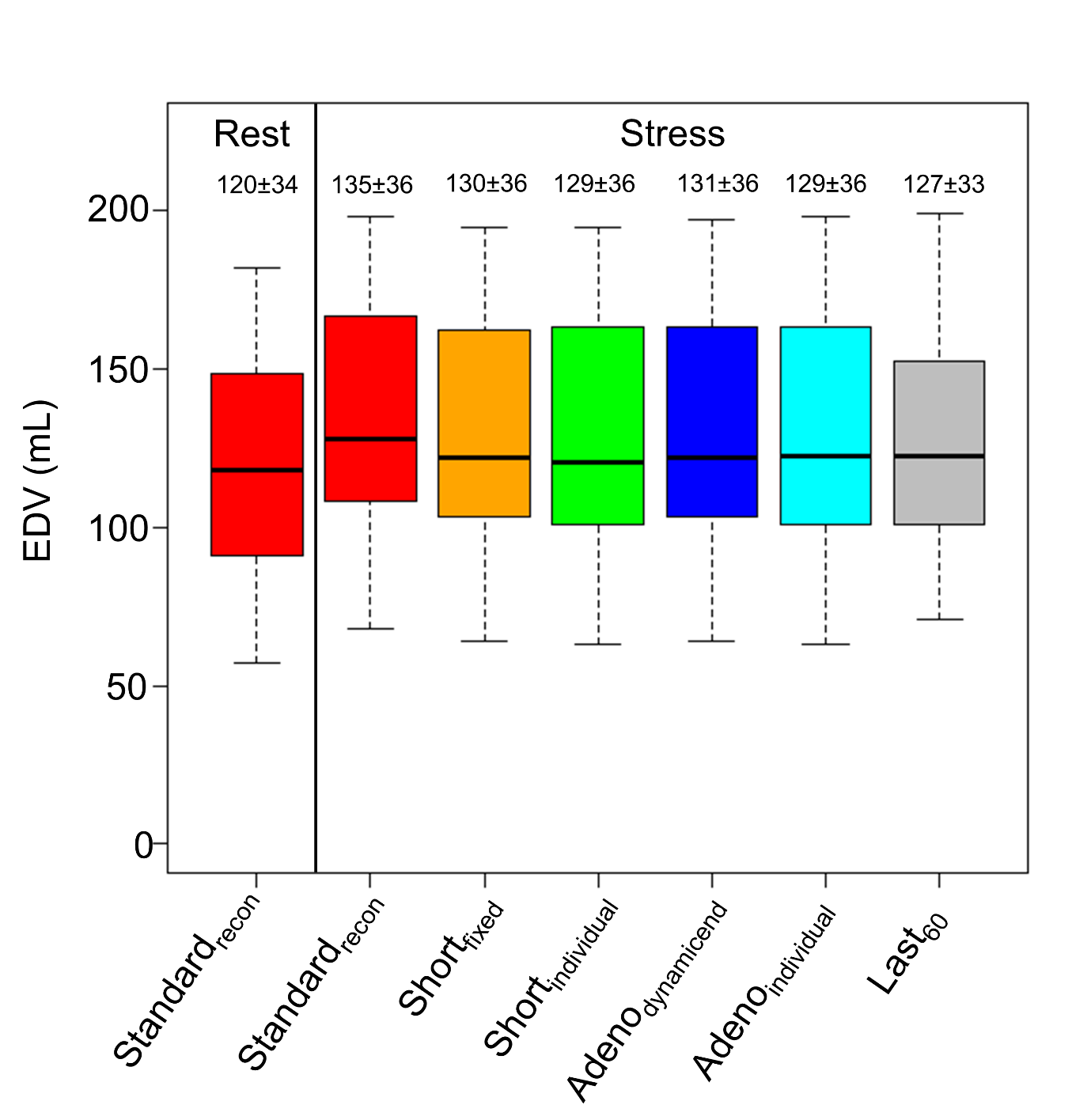
**

EDV = End-Diastolic volume.
