# Supplementary material for: Optimization of the left ventricle ejection fraction estimate obtained during cardiac adenosine stress 82Rubidium-PET scanning: impact of different reconstruction protocols
Source: J Nucl Cardiol. 2022 Apr 12;29(6):3369–78. doi: 10.1007/s12350-022-02946-1 (PMC9834342; doi:10.1007/s12350-022-02946-1)
Supplement: Supplementary file 2 — Supplementary file2 (PPTX 293 kb) [file 12350_2022_2946_MOESM2_ESM.pptx]

## Slide 1
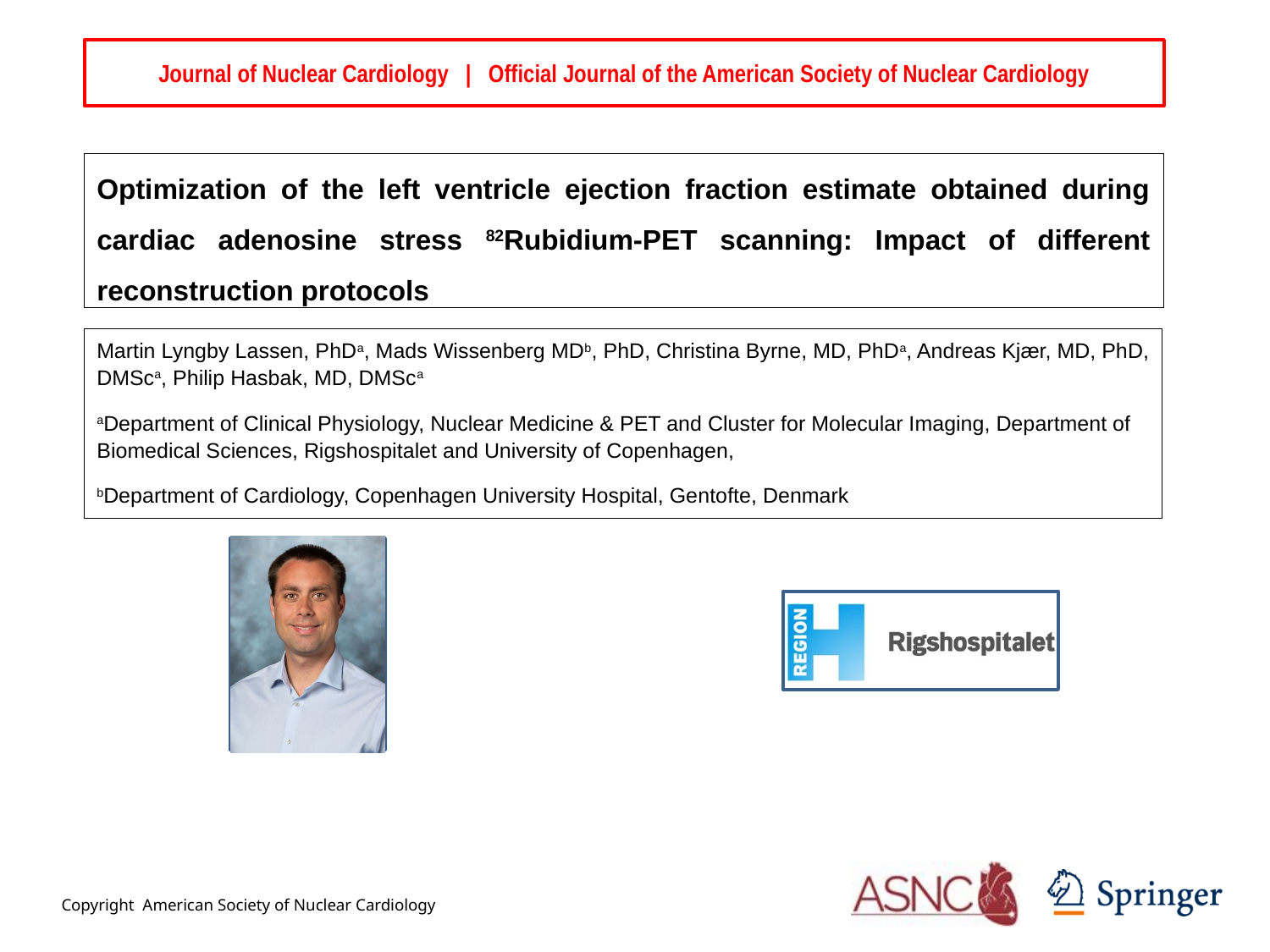

Journal of Nuclear Cardiology | Official Journal of the American Society of Nuclear Cardiology
# Optimization of the left ventricle ejection fraction estimate obtained during cardiac adenosine stress 82Rubidium-PET scanning: Impact of different reconstruction protocols
Martin Lyngby Lassen, PhDa, Mads Wissenberg MDb, PhD, Christina Byrne, MD, PhDa, Andreas Kjær, MD, PhD, DMSca, Philip Hasbak, MD, DMSca
aDepartment of Clinical Physiology, Nuclear Medicine & PET and Cluster for Molecular Imaging, Department of Biomedical Sciences, Rigshospitalet and University of Copenhagen,
bDepartment of Cardiology, Copenhagen University Hospital, Gentofte, Denmark
Copyright American Society of Nuclear Cardiology

## Slide 2
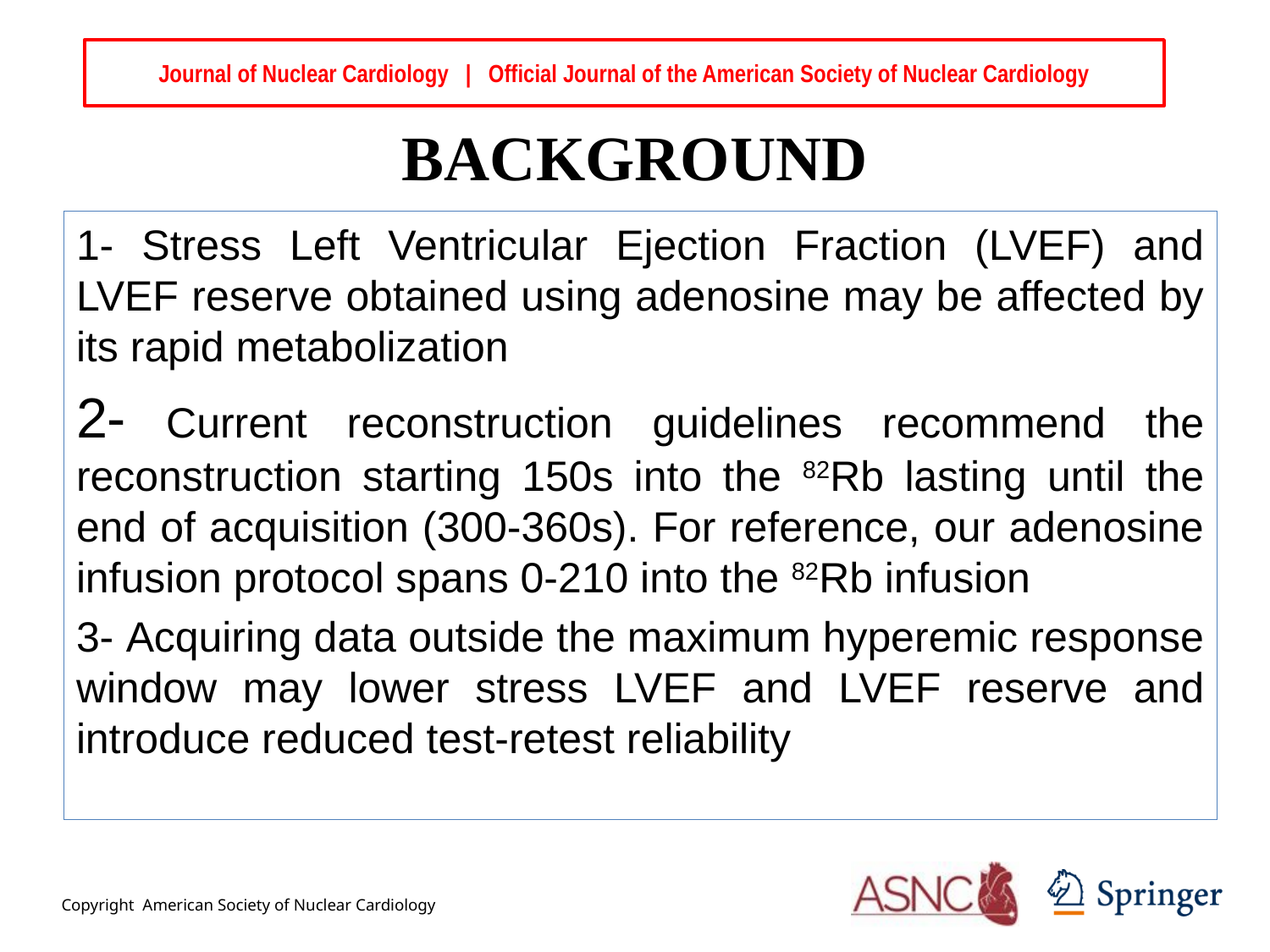

Journal of Nuclear Cardiology | Official Journal of the American Society of Nuclear Cardiology
# BACKGROUND
1- Stress Left Ventricular Ejection Fraction (LVEF) and LVEF reserve obtained using adenosine may be affected by its rapid metabolization
2- Current reconstruction guidelines recommend the reconstruction starting 150s into the 82Rb lasting until the end of acquisition (300-360s). For reference, our adenosine infusion protocol spans 0-210 into the 82Rb infusion
3- Acquiring data outside the maximum hyperemic response window may lower stress LVEF and LVEF reserve and introduce reduced test-retest reliability
Copyright American Society of Nuclear Cardiology

## Slide 3
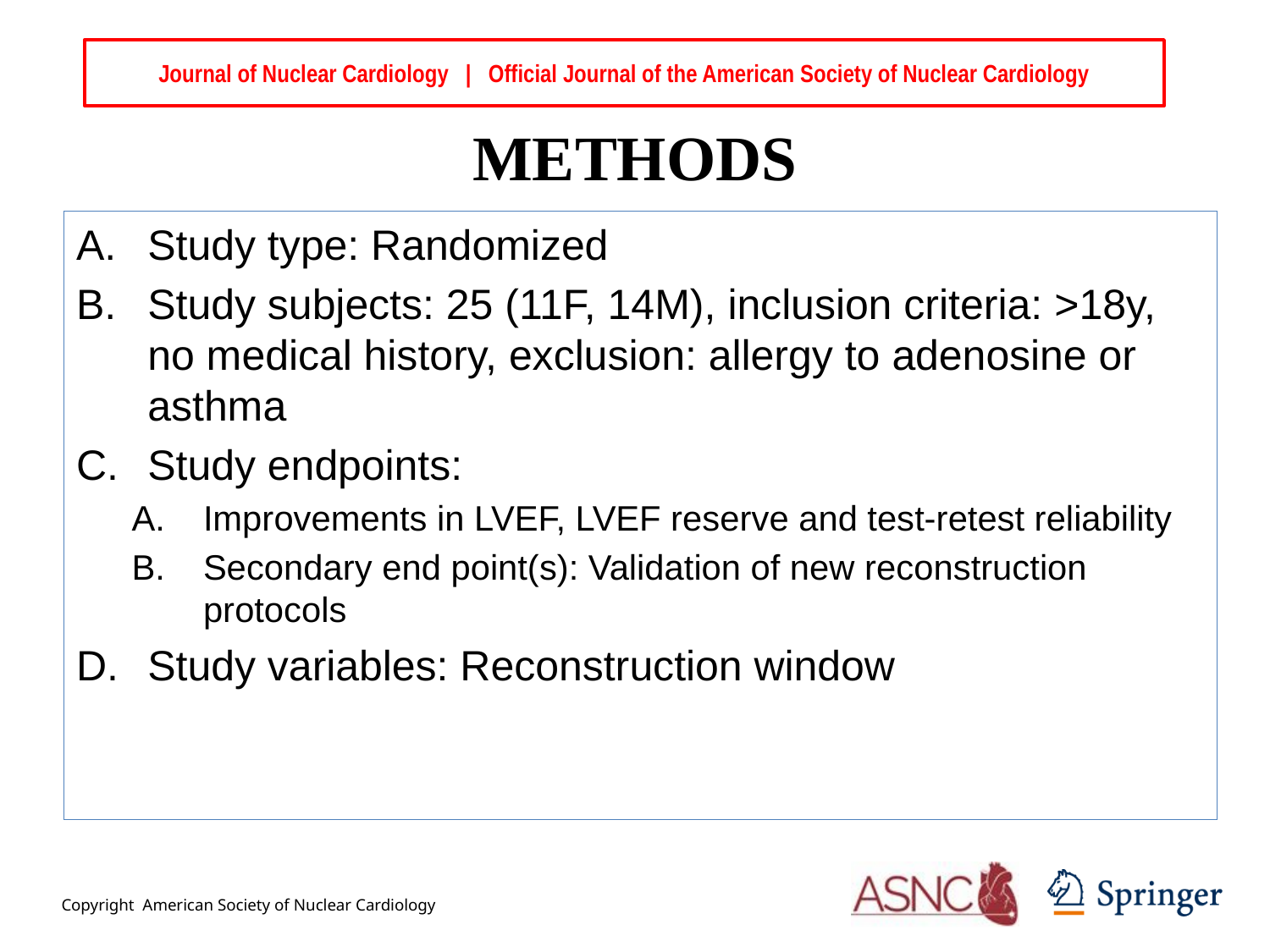

Journal of Nuclear Cardiology | Official Journal of the American Society of Nuclear Cardiology
# METHODS
Study type: Randomized
Study subjects: 25 (11F, 14M), inclusion criteria: >18y, no medical history, exclusion: allergy to adenosine or asthma
Study endpoints:
Improvements in LVEF, LVEF reserve and test-retest reliability
Secondary end point(s): Validation of new reconstruction protocols
Study variables: Reconstruction window
Copyright American Society of Nuclear Cardiology

## Slide 4
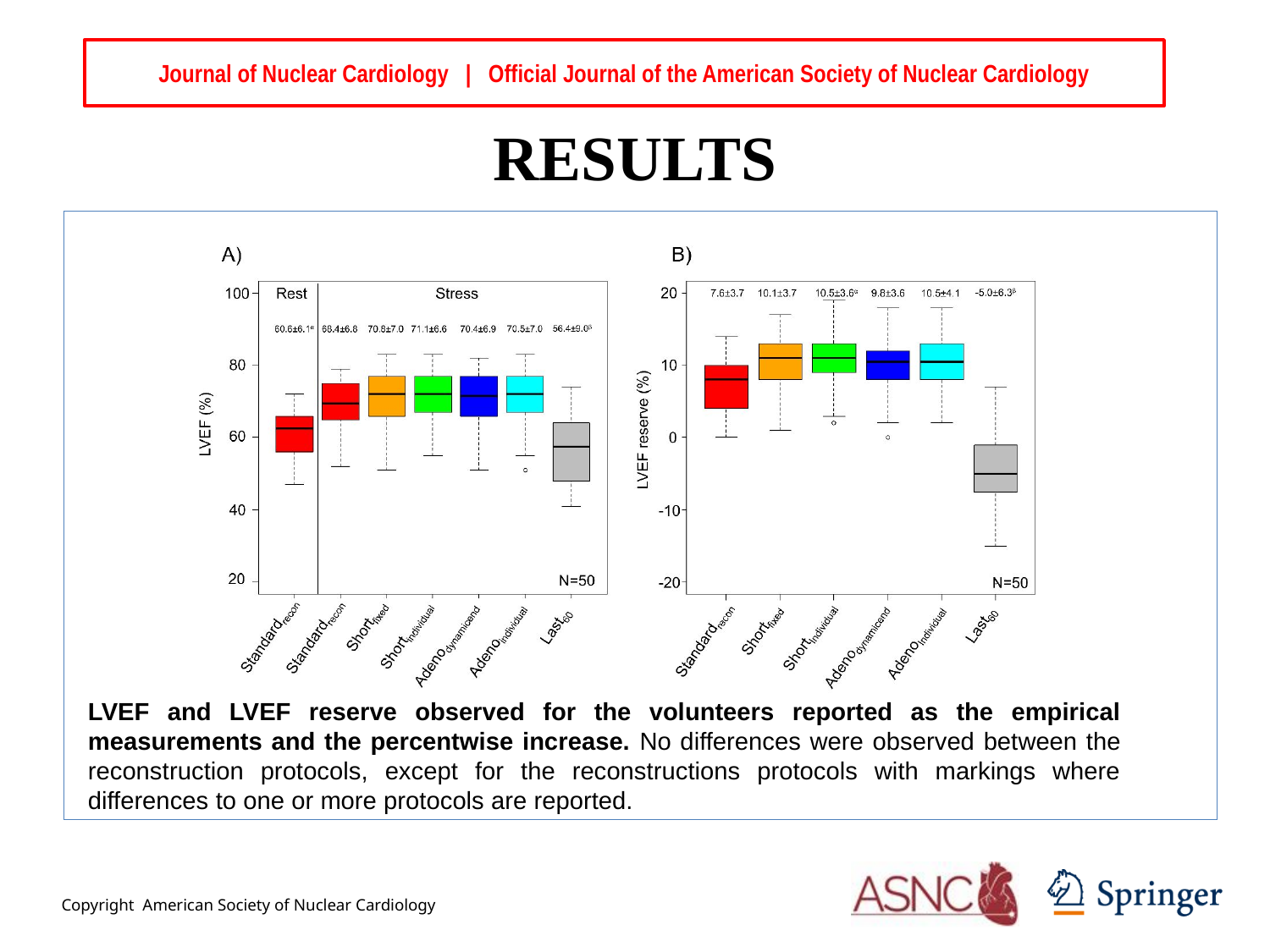

Journal of Nuclear Cardiology | Official Journal of the American Society of Nuclear Cardiology
# RESULTS
Insert a key table or a key figure
If figure, insert legend
LVEF and LVEF reserve observed for the volunteers reported as the empirical measurements and the percentwise increase. No differences were observed between the reconstruction protocols, except for the reconstructions protocols with markings where differences to one or more protocols are reported.
Copyright American Society of Nuclear Cardiology

## Slide 5
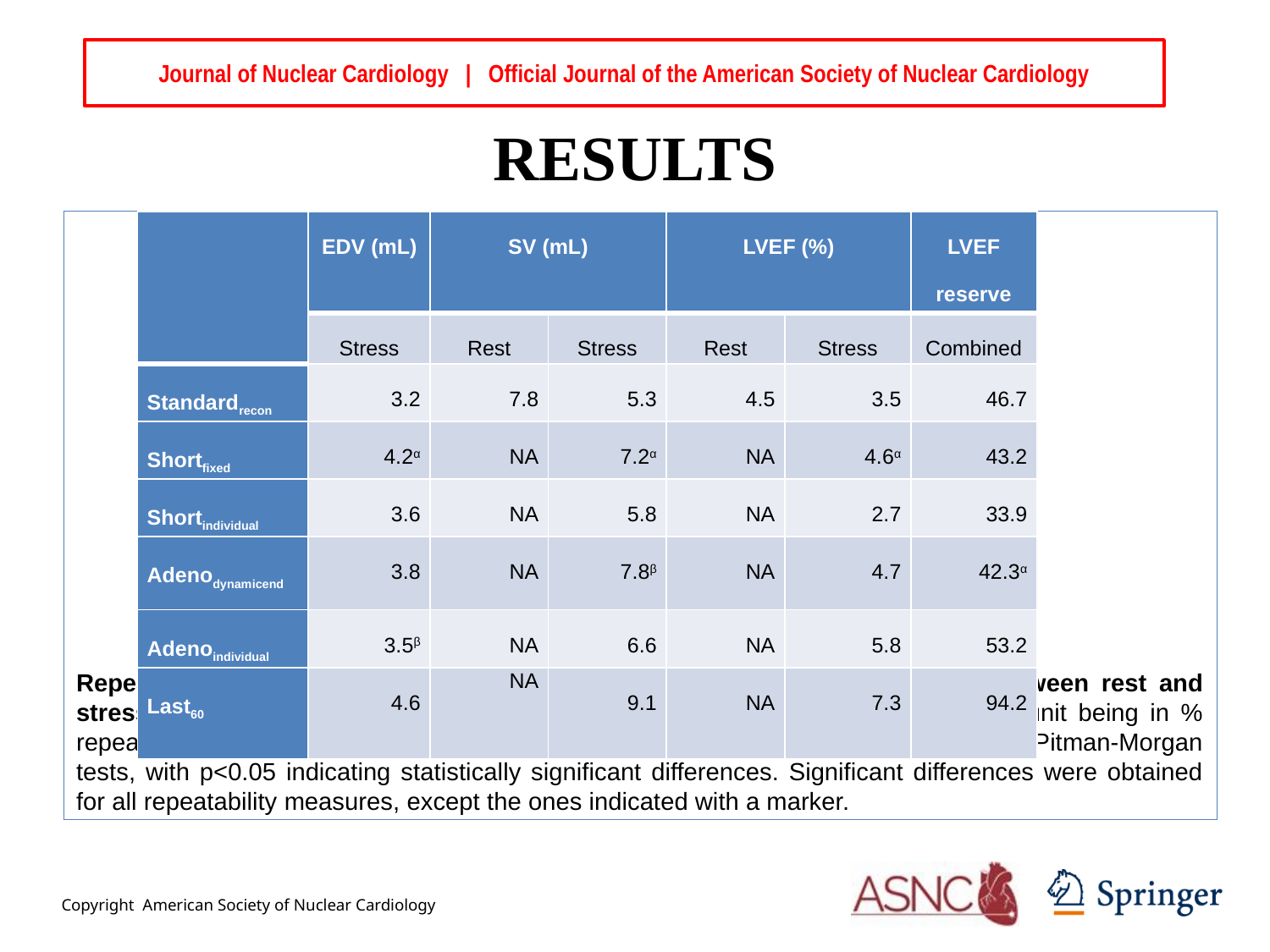

Journal of Nuclear Cardiology | Official Journal of the American Society of Nuclear Cardiology
# RESULTS
Repeatability measures obtained for SV, LVEF and percent change in EF between rest and stress MPI. Test-retest measures were obtained using the RMS method, with the unit being in % repeatability. Statistical differences in the test-retest measures were evaluated using Pitman-Morgan tests, with p<0.05 indicating statistically significant differences. Significant differences were obtained for all repeatability measures, except the ones indicated with a marker.
| | EDV (mL) | SV (mL) | | LVEF (%) | | LVEF reserve |
| --- | --- | --- | --- | --- | --- | --- |
| | Stress | Rest | Stress | Rest | Stress | Combined |
| Standardrecon | 3.2 | 7.8 | 5.3 | 4.5 | 3.5 | 46.7 |
| Shortfixed | 4.2α | NA | 7.2α | NA | 4.6α | 43.2 |
| Shortindividual | 3.6 | NA | 5.8 | NA | 2.7 | 33.9 |
| Adenodynamicend | 3.8 | NA | 7.8β | NA | 4.7 | 42.3α |
| Adenoindividual | 3.5β | NA | 6.6 | NA | 5.8 | 53.2 |
| Last60 | 4.6 | NA | 9.1 | NA | 7.3 | 94.2 |
Copyright American Society of Nuclear Cardiology

## Slide 6
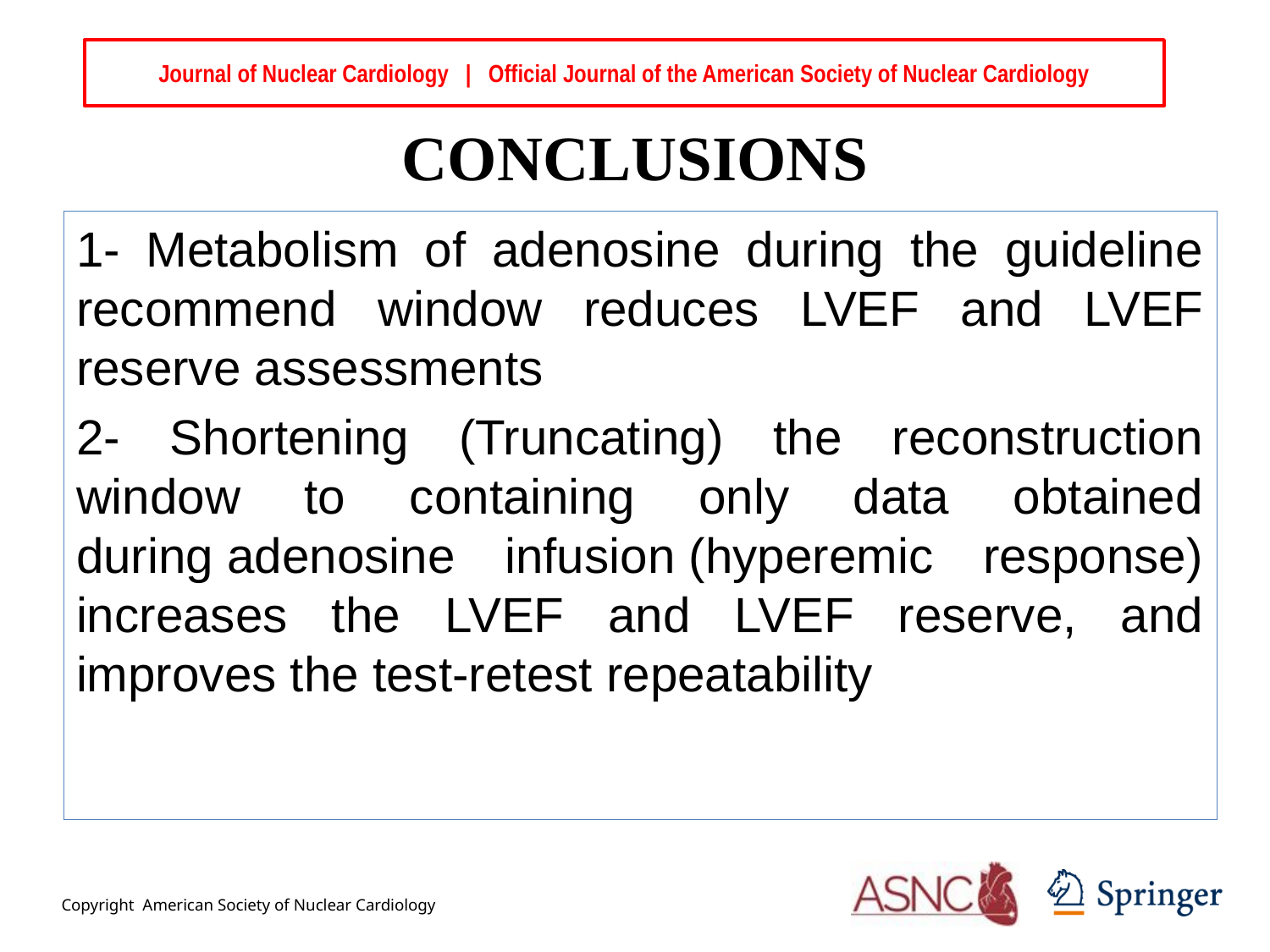

Journal of Nuclear Cardiology | Official Journal of the American Society of Nuclear Cardiology
# CONCLUSIONS
1- Metabolism of adenosine during the guideline recommend window reduces LVEF and LVEF reserve assessments
2- Shortening (Truncating) the reconstruction window to containing only data obtained during adenosine infusion (hyperemic response) increases the LVEF and LVEF reserve, and improves the test-retest repeatability
Copyright American Society of Nuclear Cardiology
