# Supplementary figures and images for: Optimization of the left ventricle ejection fraction estimate obtained during cardiac adenosine stress 82Rubidium-PET scanning: impact of different reconstruction protocols
Source: J Nucl Cardiol. 2022 Apr 12;29(6):3369–78. doi: 10.1007/s12350-022-02946-1 (PMC9834342; doi:10.1007/s12350-022-02946-1)

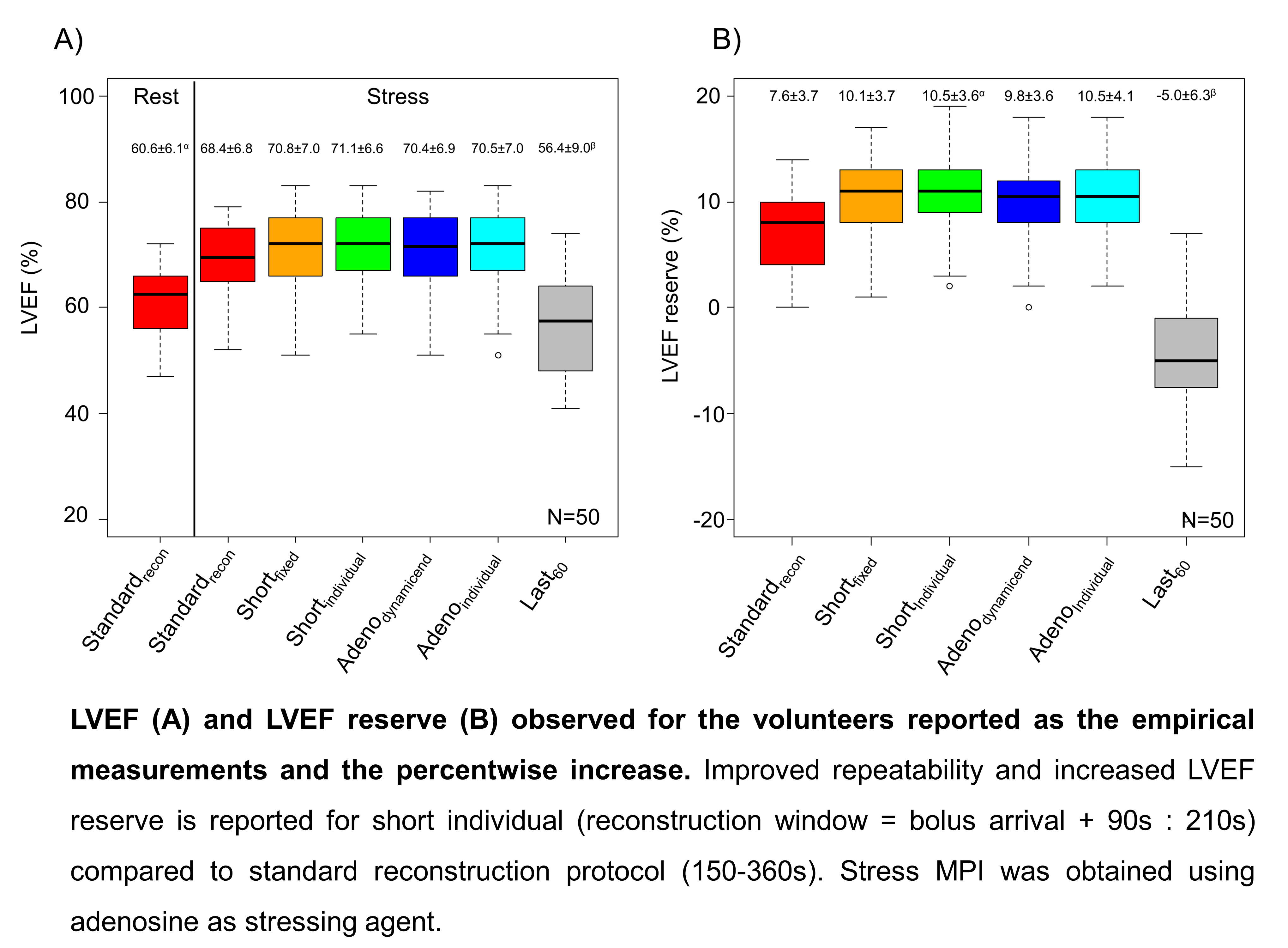

Supplement: Supplementary file 3 — Supplementary file3 (TIF 1263 kb) [file 12350_2022_2946_MOESM3_ESM.tif]
